# Immunoadjuvant Therapy and Noninvasive Ventilation for Acute Respiratory Failure in Lung Tuberculosis: A Case Study

**DOI:** 10.1155/2015/283867

**Published:** 2015-07-27

**Authors:** René Agustín Flores-Franco, Dahyr Alberto Olivas-Medina, Cesar Francisco Pacheco-Tena, Jorge Duque-Rodríguez

**Affiliations:** ^1^Departamento de Medicina Interna, Christus Muguerza Hospital Del Parque, Calle Dr. Pedro Leal Rodriguez 1802, Colonia Centro, 31000 Chihuahua, CHIH, Mexico; ^2^Facultad de Medicina, Universidad Autónoma de Chihuahua, Circuito Universitario Campus II, 31240 Chihuahua, CHIH, Mexico; ^3^Servicios de Salud de Chihuahua, Sistema Estatal de Salud, Calle Tercera No. 604, Piso 3 Colonia Centro, 31000 Chihuahua, CHIH, Mexico

## Abstract

Acute respiratory failure caused by pulmonary tuberculosis is a rare event but with a high mortality even while receiving mechanical ventilatory support. We report the case of a young man with severe pulmonary tuberculosis refractory to conventional therapy who successfully overcame the critical period of his condition using noninvasive ventilation and immunoadjuvant therapy that included three doses of etanercept 25 mg subcutaneously. We conclude that the use of etanercept along with antituberculosis treatment appears to be safe and effective in patients with pulmonary tuberculosis presenting with acute respiratory failure.

## 1. Introduction

In 2013, 9 million people became sick with tuberculosis and there were approximately 1.5 million tuberculosis-related deaths worldwide [1]. Acute respiratory failure (ARF) is considered an unusual complication of pulmonary tuberculosis (PT) with an estimated incidence of 1.5% in hospitalized patients and a mortality of 69%, mainly in cases that require mechanical ventilation [2]. Respiratory failure associated with PT may present with an acute form of a disease, such as miliary tuberculosis, acute respiratory distress syndrome (ARDS), and bronchopneumonia, or chronically as a consequence of respiratory, musculoskeletal, or surgical sequels [3]. Conventional treatment of acute disease includes immediate antituberculosis chemotherapy and, if required, mechanical ventilation. Noninvasive pressure support ventilation (NIPSV) and other adjuvant therapies have been useful and provided variable results [4–9]. Here, we present the case of a young patient with ARF, secondary to PT who particularly benefited from a treatment that included these two modalities.

## 2. Case Report

**Figure 1:**
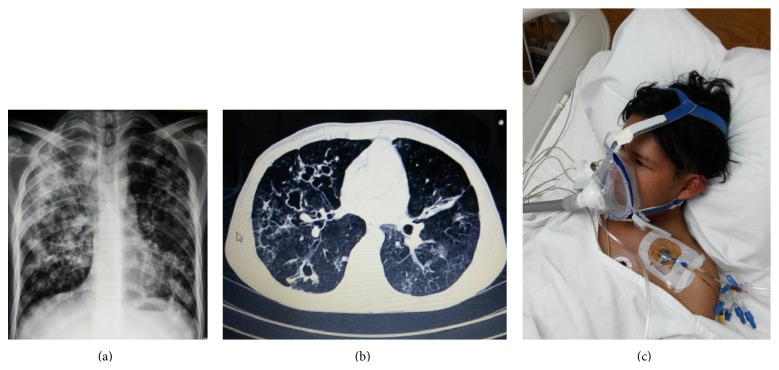
(a) Chest radiograph showing extensive multifocal consolidation and cavitation predominantly in the right upper lobe. (b) Computed tomography (CT) image scan obtained 5 weeks later shows the persistence of some caverns, nodules, and linear opacities but a significant improvement in areas of consolidation. (c) An oronasal mask was used to minimize air leakage and improve tolerance for noninvasive ventilation. Health personnel should not overlook the risk of tuberculosis transmission associated with short distances exposures.

**Figure 2:**
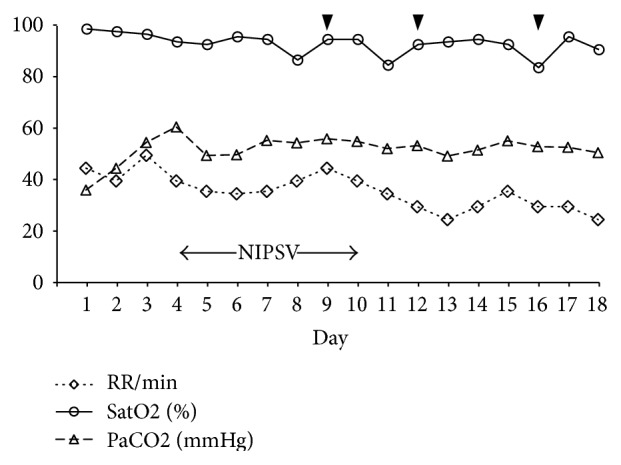
Changes in SatO2 and PaCO2 and respiratory rate (RR) in relation to the application of noninvasive pressure support ventilation (NIPSV) and 3 doses of etanercept 25 mg administered subcutaneously (black arrowheads).

A 21-year-old Tarahumara male was transferred from his community hospital with a 4-month history of cough, hemoptysis, progressive dyspnea, intermittent fever, and significant weight loss. On admission, he presented with a bad general condition, with the following vital signs: blood pressure of 90/60 mmHg, heart rate of 140 bpm, respiratory rate of 35 breaths per minute, and core body temperature of 99.5°F. The physical examination revealed a cachectic young man with evident signs of ARF including tachypnea, breathy speech, and accessory muscle use. Chest auscultation evidenced fine inspiratory crackles, mainly in the right apex. Arterial blood-gas (ABG) analysis while he breathed supplemental oxygen via a mask showed a pH of 7.37, PaO2 of 98 mmHg, PaCO2 of 36.5 mmHg, and HCO_3_^−^ of 20.8 mEq/L. Laboratory admission tests showed Hb of 11.1 g/dL, white blood count (WBC) of 11.6 cells/*μ*L, neutrophils count of 10.9/*μ*L, lymphocytes count of 0.2/*μ*L, Na^+^ of 136 mmol/L, Cl^−^ of 98 mmol/L, K^+^ of 4.02 mmol/L, Ca^2+^ of 7.6 mg/dL, glucose of 77 mg/dL, Cr of 0.36 mg/dL, blood urea nitrogen (BUN) of 6.1 mg/dL, uric acid of 3.7 mg/dL, cholesterol of 91 mg/dL, triglycerides of 98 mg/dL, and albumin of 2.1 g/dL. The HIV and hepatitis B and C tests were all negative. Sputum acid-fast stains were positive since his previous hospitalization and a real-time polymerase chain reaction (PCR) assay performed with another sputum sample confirmed the presence of* Mycobacterium tuberculosis* DNA. A chest X-ray showed diffuse alveolar and nodular opacities, as well an extensive right upper lobe cavitary disease (Figure 1). Based on the above findings, we calculated an APACHE II score of 13. The patient was treated with hydrocortisone 100 to 250 mg intravenously for 8 hours, and a daily regimen of intravenous amikacin 750 mg, and moxifloxacin 400 mg, along with antituberculosis treatment of 3 tablets of a fixed-dose combination (DoTBal, SILANES Laboratories) of rifampicin 150 mg, isoniazid 75 mg, pyrazinamide 400 mg, and ethambutol 300 mg. The patient was admitted to the intensive care unit but on day 4 in the hospital, the increased work of breathing required the initiation of NIPSV with a single-limb-circuit bilevel ventilator (VPAP III, ResMed) through an oronasal interface at pressures of 8–12/4 cm H_2_O. The DoTBal dose was increased to 4 tablets per day; however, the characteristic red color of the urine produced by rifampicin was no longer observed and the serum levels in a random sample were undetectable. Over the next 4 days despite slight improvement in PaCO2, it was not possible to wean the patient from NIPSV due to the persistent tachypnea. After a discussion regarding alternative therapies and under the respective observations of the local board of pharmacovigilance, the medical team decided as an extraordinary measure to administer etanercept (Enbrel, Wyeth Laboratories) 25 mg subcutaneously. The following day the patient showed a general improvement and an improved respiratory condition (Figure 2). After 2 days, he could finally be separated from NIPSV and undergo continued care in an isolated hospital ward breathing supplemental oxygen via nasal prongs. Three days after the first dose of etanercept, a second dose was administered without significant changes in the clinical condition of the patient. However, 4 days after the second dose of etanercept, the patient experienced exacerbation of respiratory symptoms, malaise, and fever of 100.5°F (Figure 2). Due to the short half-life of etanercept, this scenario was attributed to a paradoxical reaction and resolved promptly with the administration of a final third dose of etanercept along with hydrocortisone 200 mg intravenously. Within a few days, the clinical condition of the patient allowed his transfer to a unit with long-term care facilities, and after a month with negative smears for acid-fast bacilli he was finally discharged to their community under a directly observed therapy (DOT) program.

## 3. Discussion

While advanced disease has become less common due to the availability of treatment, our ethnic minority group remains susceptible to advanced presentations of tuberculosis owing to recognized factors such as poverty, malnutrition, alcoholism, cultural aspects, and lack of access to health services [10]. In these patients, respiratory failure is explained by the dissemination of infection which leads to pneumonia, cavitation, miliary spread, lobar collapse, pneumothorax, or pleural effusion. The mortality of patients with active pulmonary tuberculosis who require mechanical ventilation is high and their most common indications occur in patients with ARDS, widespread fibrocavitary disease, and pneumonia.

In patients who are treated with regimens including rifampicin and isoniazid combination, a normalization of gas exchange is usually observed within 3-4 weeks; however, in some cases, normalization may not occur for months or never be achieved due to the extent of the sequels [11]. In Mexico, first-line antituberculosis drugs are only available as oral rather than intravenous preparations. Unfortunately, in patients with severe tuberculosis oral administration does not guarantee therapeutic levels of drug due to poor absorption as a consequence of a decreased gastric emptying time and decreased functional absorptive area in the intestines. This decreased absorption influences the pharmacokinetics and may contribute to the subtherapeutic plasma concentrations of these drugs, especially rifampin [12]. This argument is used to justify the use of the parenteral route of antituberculosis drugs, such as aminoglycosides, quinolones, and linezolid in critically ill tuberculosis patients [13].

Additionally, NIPSV has also been shown to be useful in patients with pulmonary tuberculosis. NIPSV may be indicated in patients with chronic respiratory failure due to severe sequels of tuberculosis and during exacerbations, such as in cases of COPD [3, 14]. However, the role of NIPSV in acute respiratory failure in patients with pulmonary tuberculosis is currently a debatable issue because some of these cases have recovered only with drug therapy. Recently, Agarwal et al. successfully used NIPSV for periods of 5–10 days in 3 patients with active pulmonary tuberculosis presenting nonhypercapnic respiratory failure and metabolic acidosis [4]. However, Valade et al. reported that among 53 patients with tuberculosis who were admitted to the intensive care unit, 27 (51%) required mechanical ventilation. Of these patients, 8 (15%) were initially treated with NIPSV but all of them eventually required tracheal intubation [5].

Granulomas are the hallmark of the host response to mycobacteria and represents bacteriostatic efforts to physically contain an infection that cannot be otherwise controlled by host defenses. The microenvironment generated inside the granuloma affects the metabolism, biosynthesis, and replication of the mycobacteria resulting in its semidormant state for prolonged periods. However, the granuloma may be associated with adverse effects on the host. During granuloma formation, initially infected macrophages die; the recruited macrophages then phagocytose the infected cell remnants and their bacterial contents promote the expansion of the mycobacteria population [15]. The physical effect as a barrier and the microenvironment generated within the granuloma reduce the bactericidal capacity of antituberculosis drugs, especially isoniazid [16]. Furthermore, the fully organized granuloma displaces parenchymal tissue and causes the development of perigranulomatous fibrosis, resulting in tissue damage [17]. The application of corticosteroids in patients with tuberculosis, especially in those with an extrapulmonary presentation of the disease, may inhibit the nonselective release of lymphokines and cytokines responsible for constitutional symptoms and tissue damage produced by granulomas [18]. In addition, disruption of the granuloma integrity by corticosteroids enhances the penetration of antituberculosis drugs [16, 19]. The beneficial effects of corticosteroids in patients with pulmonary tuberculosis and acute respiratory failure have been reported in cases of miliary tuberculosis and ARDS [20]. Nevertheless, corticosteroid therapy with tuberculosis remains controversial because of the retrospective nature of the research conducted to date. Therefore, recommendations should be established on an individual basis.

Tumor necrosis factor-alpha (TNF-*α*) is a potent proinflammatory cytokine that is expressed by macrophages and T cells and is considered essential for the formation and maintenance of granuloma [17]. Additionally, it is responsible for the systemic inflammatory response that is manifested by constitutional symptoms, including cachexia. Excessive production of TNF-*α* in active tuberculosis could contribute to tissue damage and lower concentrations, as observed in latent tuberculosis, would be responsible for maintaining the integrity of the granuloma, thus inhibiting the growth and spread of the mycobacteria [21]. In experimental models, the selective neutralization of TNF-*α* inhibits the formation of granulomas and reduces the microbicidal activity of macrophages and NK cells [16]. This would explain why patients with rheumatic diseases treated with an anti-TNF-*α* inhibitor have an increased risk of activation and dissemination of latent tuberculosis. The recognition of the important role of TNF-*α* in maintaining a semidormant state of mycobacteria has led to the development of screening and treatment guidelines for latent tuberculosis infection in patients receiving anti-TNF-*α* agents [22].

The role of anti-TNF-*α* therapy in cases of active tuberculosis is still subject to discussion. On the one hand, there is the disputed situation in which immunity is required for sterilization of the mycobacterial infection and delayed resolution of the granulomatous host response may adversely affect the resolution of infection [6, 7]. On the other hand, anti-TNF-*α* therapy has demonstrated an anecdotal prompt and beneficial effect in controlling steroid-resistant tuberculosis paradoxical reactions that occur after initiating antituberculosis treatment for severe tuberculosis with or without prior exposure to anti-TNF-*α* therapy [8, 9]. A paradoxical reaction in a patient with tuberculosis is defined as a worsening of the patient's conditions after initiation of antituberculous therapy and is attributed to the phenomenon of immunorestitution. The usefulness of anti-TNF-*α* therapy has also been demonstrated in two prospective studies that included HIV patients with newly diagnosed tuberculosis in whom an anti-TNF-*α* inhibitor accelerated the response to tuberculosis treatment [23, 24]. In those studies, patients received 8 doses of etanercept 25 mg subcutaneously twice weekly, starting on day 4 of the tuberculosis therapy. Similarly, as described for the use of corticosteroids, anti-TNF-*α* therapy may contribute to the response to tuberculosis therapy allowing the penetration of drugs into granulomas or improving their bactericidal activity against metabolically active bacilli. Unlike these studies, we did not seek to accelerate the sputum culture conversion or improve the medium and long-term prognosis. Our primary objective was to save the life of the patient and avoid possible therapeutic maneuvers with high risk of complications and significant mortality such as mechanical ventilation.

## 4. Conclusions

Mechanical ventilation in critically ill patients with PT is associated with a high mortality and thus studies are required to examine the benefits of NIPSV and immunomodulatory adjuvant treatment. Etanercept, which is associated with tuberculosis treatment, seems to be effective and safe in patients with active tuberculosis and should be seriously considered for use in patients with respiratory failure.

## Abbreviations

ARF: Acute respiratory failure
PT: Pulmonary tuberculosis
ARDS: Acute respiratory distress syndrome
NIPSV: Noninvasive pressure support ventilation
ABG: Arterial blood-gas
BUN: Blood urea nitrogen
WBC: White blood count
PCR: Polymerase chain reaction
DOT: Directly observed therapy
TNF-*α*: Tumor necrosis factor-alpha.
